# Whither life? Conjectures on the future evolution of biochemistry

**DOI:** 10.1098/rsbl.2016.0269

**Published:** 2016-08

**Authors:** Jodi L. Brewster, Thomas J. Finn, Miguel A. Ramirez, Wayne M. Patrick

**Affiliations:** Department of Biochemistry, University of Otago, Dunedin, New Zealand

**Keywords:** carbon fixation, energy generation, genetic circuits, CRISPR/Cas, phenotypic novelty

## Abstract

Life has existed on the Earth for approximately four billion years. The sheer depth of evolutionary time, and the diversity of extant species, makes it tempting to assume that all the key biochemical innovations underpinning life have already happened. But we are only a little over halfway through the trajectory of life on our planet. In this Opinion piece, we argue: (i) that sufficient time remains for the evolution of new processes at the heart of metabolic biochemistry and (ii) that synthetic biology is providing predictive insights into the nature of these innovations. By way of example, we focus on engineered solutions to existing inefficiencies in energy generation, and on the complex, synthetic regulatory circuits that are currently being implemented.

## 1. Introduction

**Figure 1.**
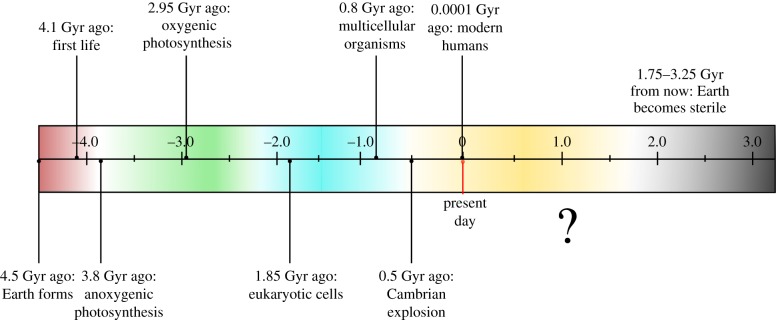
The complete evolutionary trajectory of life on the Earth, with the timeline (in Gyr) drawn to scale. Selected innovations from the past are shown and the yellow shading highlights the time remaining for future innovations to occur.

The Earth appeared out of stardust as a tangible mass approximately 4.5 × 10^9^ years (4.5 Gyr) ago [1]. Life arose relatively rapidly, with recent evidence suggesting that the first biogenic signatures in the geological record may be as old as 4.1 Gyr [2]. Since then, species have evolved to colonize almost every conceivable niche on our planet. Major evolutionary innovations (figure 1) have included the emergence of eukaryotes, of multicellularity (at least 25 times; [3]), and of complex animal body plans during the Cambrian explosion. These organism-level innovations have been enabled by the evolution of molecular processes such as the ability to generate ATP by glycolysis and oxidative phosphorylation, to fix atmospheric CO_2_ via oxygenic photosynthesis, and to regulate gene expression with exquisite spatial and temporal control. In many cases, enzyme-catalysed metabolic pathways are likely to have arisen from non-enzymatic reaction sequences [4], as chemistry was harnessed by biology. In the light of such wondrous biochemistry, it is easy to assume that all the major transitions in evolution [5] have already occurred, and that many key metabolic processes are sufficiently fit for purpose that they are therefore essentially immutable.

In this article, we seek to update this view. It will be another 1.75–3.25 Gyr before the expansion of the sun causes the surface temperature of the Earth to soar, the atmosphere to disintegrate and our planet to become sterile [6]. We are only 55–70% of the way through the evolutionary trajectory of life on the Earth (figure 1). With this in mind, our central thesis is that new biochemical innovations, on the scale of photosynthesis or glycolysis, will continue to emerge in the biosphere and to become as central to biology as these processes are currently. To wit, we opine that evolution to date has under-sampled the possible solutions to biochemical problems. Further, we suggest that the field of synthetic biology should be viewed as ‘biology not yet in the databases’. The solutions of synthetic biologists to biochemical challenges may have existed in the past, may exist (but remain undiscovered) in the biosphere today—or of most relevance for our discussion, they may evolve in the future (by natural pathways, rather than via escape of synthetic genes or organisms from the laboratory). In particular, we note that horizontal gene transfer is so ubiquitous [7], and evolutionary timescales are so long that the heterologous gene combinations currently being explored by metabolic engineers are also likely to be sampled naturally in the future. In the sections below, we highlight examples in which synthetic biology may have already (perhaps unwittingly) informed the future of biochemical evolution.

## 2. Enhancements to carbon fixation and energy generation

Energy generation and conservation are central components of an organism's evolutionary fitness. The following examples demonstrate ways in which synthetic biology is extending natural biochemical processes beyond what evolution has produced to date.

Carbon enters the biosphere via photosynthesis. Even after billions of years of evolution (figure 1), aspects of this process remain woefully inefficient. For example, land plants only absorb light of wavelengths 400–700 nm, corresponding to approximately 50% of total incident solar energy. Strategies for engineering plants that use more of the solar spectrum have been proposed [8,9], although these are yet to be implemented by synthetic biologists. The restricted wavelength range used by land plants is, in part, due to their reliance on chlorophylls *a* and *b* for light absorption. Diverse cyanobacteria harvest light over the range 350–1075 nm, due to photosynthetic reaction centres that contain various bacteriochlorophylls [9]. Land plants will effectively double their solar photon capture if they evolve to use hybrid photosynthetic systems that incorporate bacterial reaction centres.

The enzyme ribulose-1,5-bisphosphate carboxylase/oxygenase (Rubisco) catalyses the incorporation of CO_2_ into biomass. Rubisco displays poor kinetic parameters and wastefully accepts O_2_ as a substrate that competes with CO_2_. Circumventing these undesirable properties in the most affected plants could improve their photosynthetic efficiency [9], although attempts to achieve this by engineering the enzyme have met with limited success so far. This is perhaps unsurprising, given the structural and chemical similarities of CO_2_ and O_2_. An alternative solution, used by cyanobacteria, is to concentrate CO_2_ in Rubisco-containing organelles called carboxysomes. A functional cyanobacterial Rubisco has recently been expressed within carboxysome-like structures, in the chloroplasts of engineered tobacco plants. While the cyanobacterial enzyme was expressed at a lower level than the native tobacco Rubisco, it showed higher rates of CO_2_ fixation per unit of enzyme [10]. Rather than Rubisco, archaea in the phylum Thaumarchaeota use a bifunctional acetyl-CoA/propionyl-CoA carboxylase to fix CO_2_ under aerobic conditions, but without a competing oxygenase reaction [11]. Plausible, artificial carbon fixation pathways that use entirely novel combinations of metabolic enzymes, and that are predicted to be more efficient than the predominant natural pathway (the Calvin cycle), have also been proposed [12]. These design efforts emphasize the numerous routes by which selection may act to effect wholesale changes in the process of CO_2_ fixation, particularly if the phylogenetic distribution of efficient, Thaumarchaeota-type carboxylases expands in the distant future.

In anaerobic environments, fermentation is essential for converting sugars to acids, alcohols or gases. For many microorganisms, it is often the only source of mass and energy. However, most organisms are extremely limited in the range of carbon sources they can ferment. These limited substrate ranges are readily overcome by metabolic engineering. For example, the yeast *Saccharomyces cerevisiae* has been engineered to use three components of lignocellulosic biomass (cellobiose, xylose and acetic acid) simultaneously [13]. This was achieved through a combination of serial passaging and heterologous expression of enzymes from other microorganisms, and thus mimics natural processes of growth under selection and horizontal gene transfer. Further, some obligately anaerobic, thermophilic bacteria produce multi-enzyme complexes termed cellulosomes, which are anchored to the exterior surface of the cell and mediate the efficient degradation of plant biomass [14]. *Saccharomyces cerevisiae* has been engineered to display functional cellulosomes on its surface, enabling the direct conversion of cellulose to ethanol [15]. Multi-substrate fermentation pathways and extracellular structures such as cellulosomes would dramatically alter niche availability for a facultatively anaerobic mesophile, such as *S. cerevisiae*, were they to be acquired.

**Figure 2.**
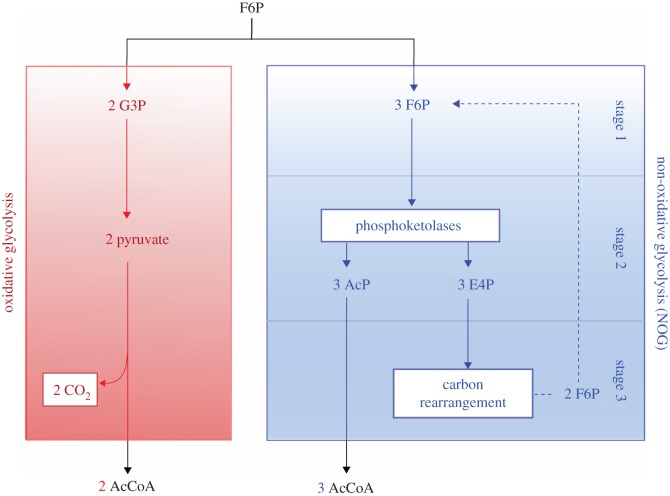
A comparison of natural (oxidative) glycolysis and synthetic (non-oxidative) glycolysis. Abbreviations are defined in the text. Redrawn from [16].

A number of glycolytic pathways have evolved for the oxidation and splitting of 6-carbon sugars to yield two molecules of 3-carbon pyruvate. For downstream processes such as biosynthesis and ATP generation via oxidative phosphorylation, these pyruvate molecules are converted to acetyl-CoA, with concomitant loss of CO_2_. Thus, only four carbons from the original hexose are used by the cell. This fundamental inefficiency has been circumvented through the design and implementation of a pathway dubbed non-oxidative glycolysis (NOG) in *Escherichia coli*, which allows complete conversion of a hexose to three molecules of acetyl-CoA [16]. In this cyclic pathway (figure 2), one input molecule of fructose 6-phosphate (F6P) is combined with two additional F6P molecules, and converted to three molecules of acetyl phosphate (AcP) plus three molecules of erythrose 4-phosphate (E4P) by phosphoketolase enzymes. The three E4Ps are rearranged to regenerate the two F6Ps that were initially invested, while the acetyl phosphate can enter 2-carbon metabolism. The NOG pathway facilitates complete carbon conservation but does not generate any reducing equivalents. Essential intermediates from oxidative glycolysis, such as phosphoenolpyruvate (which drives the phosphotransferase sugar uptake system), are also absent from the NOG pathway. Thus, it would not be trivial to replace glycolysis entirely with NOG. On the other hand, an organism that evolved to express and regulate both pathways would have a new ability to economize its resources by managing carbon flux and minimizing wasteful CO_2_ production.

## 3. The future of gene regulation

Much of synthetic biology is concerned with designing complicated genetic circuits that allow logical programming to be executed in living cells. From an evolutionary perspective, advances in gene regulation have been critical for innovations such as multicellularity, the emergence of complex developmental pathways, and the rise of morphological diversity in animals [17]. The evolution of new regulatory logic is certain to underpin dramatic phenotypic changes in future lineages.

Natural ligand-responsive transcription factors can be readily incorporated into new regulatory contexts. For example, an engineered pathway for biodiesel production was improved dramatically by introducing a dynamic sensor-regulator system, which continuously monitored the level of a key intermediate (fatty acyl-CoA) and controlled flux through the pathway accordingly [18]. Using the LuxR transcriptional activator, which responds to cell-permeable acyl-homoserine lactones, it has also been possible to engineer a population of *E. coli* cells that display synchronized oscillations in their patterns of gene expression [19]. These examples emphasize the relative ease with which new temporal, spatial and intercellular behaviours can emerge.

We are currently witnessing an explosion in innovative biotechnological applications for CRISPR/Cas technology. Bacteria use CRISPR/Cas to defend against viral infection; however, it is plausible that they will evolve to expand its use for processes such as genome maintenance and modification. For example, synthetic biologists have shown that the introduction of multiple guide RNAs (gRNAs) effectively accelerates genome evolution via simultaneous modification of multiple loci [20].

In CRISPR interference (CRISPRi), a catalytically inactive variant of the Cas9 enzyme is used to manipulate gene expression by mediating repression or activation [21]. It has rapidly emerged as a versatile system for constructing robust and orthogonal circuits. A recent example is a set of logic gates that interfaces with natural regulatory networks to transduce synthetic inputs (e.g. anhydrotetracycline) into global cellular outputs (e.g. changes to bacteriophage resistance profiles) [22]. In human cell lines, there is substantial interest in engineering cell fate. An intriguing prospect is to reprogramme differentiated cells into induced pluripotent stem cells, by controlling expression of the relevant transcription factors. The first step towards this goal—activation of the reprogramming factor OCT4—has recently been achieved [23].

Naturally occurring CRISPR/Cas-based systems that are capable of manipulating and reprogramming cell fates in higher eukaryotes may sound fanciful; however, biotechnology may also be providing insight into what will evolve over the next few billion years, simply by piecing together pre-existing cellular machinery. Indeed, multicellular organisms have evolved from unicellular ancestors in numerous lineages, and they have also reverted to unicellular states [3]. In the time remaining for life on the Earth, it is inevitable that adaptation via variation in genetic circuitry, perhaps mediated by CRISPR/Cas-like machinery, will lead to the emergence of new multicellular lineages, as well as the reversion to unicellularity of others.

## 4. Concluding remarks

Evolutionary biology seeks to explain the diversity of life on the Earth today, by inferring past events. Here, we have instead sought to focus attention on the future evolution of biochemistry. We have highlighted metabolic inefficiencies, the solutions to which are likely to confer adaptive advantages. We have also conjectured that the nascent field of synthetic biology may be offering glimpses of future evolutionary events. One highlight is the effective utilization of protein-based complexes such as carboxysomes and cellulosomes. Another is the potential for complex regulatory and developmental pathways to be implemented by new mechanisms, such as CRISPRi. We have suggested it is highly probable that these molecular innovations will arise naturally. Ultimately, however, only time will tell whether there are viable evolutionary trajectories for realizing them in ways that increase organismal fitness.
